# Multiple-infection and recombination in HIV-1 within a longitudinal cohort of women

**DOI:** 10.1186/1742-4690-6-54

**Published:** 2009-06-03

**Authors:** Alan R Templeton, Melissa G Kramer, Joseph Jarvis, Jeanne Kowalski, Stephen Gange, Michael F Schneider, Qiujia Shao, Guang Wen Zhang, Mei-Fen Yeh, Hua-Ling Tsai, Hong Zhang, Richard B Markham

**Affiliations:** 1 Department of Biology, Washington University, St Louis, Missouri, USA; 2 Division of Biological and Biomedical Sciences, Washington University, St Louis, Missouri, USA; 3 US Environmental Protection Agency, Washington, DC, USA; 4 Department of Oncology, Johns Hopkins University School of Medicine, Baltimore, Maryland, USA; 5 Department of Epidemiology, Johns Hopkins Bloomberg School of Public Health, Baltimore, Maryland, USA; 6 Department of Molecular Microbiology and Immunology, Johns Hopkins Bloomberg School of Public Health, Baltimore, Maryland, USA

## Abstract

**Background:**

Recombination between strains of HIV-1 only occurs in individuals with multiple infections, and the incidence of recombinant forms implies that multiple infection is common. Most direct studies indicate that multiple infection is rare. We determined the rate of multiple infection in a longitudinal study of 58 HIV-1 positive participants from The Women's Interagency HIV Study with a richer sampling design than previous direct studies, and we investigated the role of recombination and sampling design on estimating the multiple infection rate.

**Results:**

40% of our sample had multiple HIV-1 infections. This rate of multiple infection is statistically consistent with previous studies once differences in sampling design are taken into account. Injection drug use significantly increased the incidence of multiple infections. In general there was rapid elimination of secondary strains to undetectable levels, but in 3 cases a superinfecting strain displaced the initial infecting strain and in two cases the strains coexisted throughout the study. All but one secondary strain was detected as an inter- and/or intra-genic recombinant. Injection drug use significantly increased the rate of observed recombinants.

**Conclusion:**

Our multiple infection rate is consistent with rates estimated from the frequency of recombinant forms of HIV-1. The fact that our results are also consistent with previous direct studies that had reported a much lower rate illustrates the critical role of sampling design in estimating this rate. Multiple infection and recombination significantly add to the genetic diversity of HIV-1 and its evolutionary potential, and injection drug use significantly increases both.

## Background

Much recombination between HIV-1 subtypes has been documented [1,2]. Recombination in HIV requires infection with more than one virus at the cellular level within a single host. Jung et al. [3] reported an average of three to four distinct proviral genomes within infected spleen cells, which implies that the potential for recombination in HIV-1 is large. The documented recombination between subtypes further implies that HIV-1 infected individuals must have had multiple infections; that is, the same individual was infected by two or more strains of HIV-1 that overlapped temporally. An HIV-1 strain is a monophyletic group that is genetically differentiated from other such groups by fixed, diagnostic genetic differences. Individuals infected with two or more subtypes have been documented [4,5], thus the potential for inter-subtype recombination exists. Individuals infected with two or more strains of the same subtype have also been documented [6,7]. Taylor and Korber [8] estimated the incidence of multiple infections from detected intra-subtype recombinants as being up to 15% of all HIV-1 infections in some populations. Multiple infection rates calculated from observed inter- or intra-subtype recombinants, however, are estimates of the *cumulative *multiple infection rates over the evolutionary history of the viral strains involved [8], and this in turn can be influenced by factors other than recombination. For example, the only recombinants that can be observed in this type of analysis are those that have had some persistence over evolutionary time. If selection either favors or acts against multiple infection recombinants, the estimated multiple infection rates will be accordingly biased. Therefore, one must characterize a population of infected individuals directly to truly assess the rate and dynamics of multiple infection [8].

Previous studies on populations of infected individuals have indicated a low rate of multiple infection, ranging from 0% to 14% [9-14]. These studies vary tremendously in sample design, with sample sizes varying from 7 infected individuals to 718, with different numbers of HIV-1 samples being taken per individual, with different amounts and locations of the HIV-1 genome being surveyed genetically, and with some studies being a single cross-section of infected individuals and others longitudinal. Overall, these studies indicate a multiple infection incidence of 0.8% when weighted by sample size, a figure heavily influenced by one study [10], for which it was concluded that there was no evidence for multiple infection in 718 individuals. In those studies that distinguish between coinfection (the host was initially infected by two or more strains of HIV-1) and superinfection (an initial infection was followed by a later secondary infection), equal rates of 1.6% for coinfection and superinfection yield an overall rate of multiple infection of 3.2%. These results are an order of magnitude below the indirect estimates based on recombination analyses [1,8]. Indeed, the incidences of multiple infection were so low in some of these studies, that the authors speculated that some degree of protection may be generated against superinfection [11,13,14].

In this study we examine a longitudinal cohort of HIV-1 positive women coupled with genetic screens of the *pol *and *env *genes of HIV-1. To enhance power to detect coinfection and superinfection beyond that of the previous studies mentioned above, we executed a fully prospective longitudinal study on 58 participants, the largest sample with such a design. We examined all participants for both the *env *and *pol *genes and more sequences per visit than previous studies. From these data, we estimated the incidence of multiple infection and the impact of the risk factor of injection drug use (IDU) on multiple infection by including both IDUs and non-IDUs in our sample. We also investigated the temporal dynamics of superinfection and its evolutionary significance.

Because the phenomena of recombination and multiple-infection are strongly intertwined, another goal of our study is to examine the amount, patterns and evolutionary significance of inter- and intragenic recombination both within single infection strains and between strains in multiple-infected individuals. Most methods of recombination detection require a large number of informative sites, creating a strong bias towards detecting inter-strain recombination (particularly among inter-subtypes) versus recombination within a single strain within a single host [1]. By using an analytical technique developed specifically to detect intra-strain recombination in singly infected hosts that can yield a statistically significant inference of recombination with as few as six nucleotide differences between the parental genomes [15,16], we can examine the role of recombination at all these biological levels with much greater resolution than previous studies.

## Results

### Incidence of multiple infection, coinfection, and superinfection

Twenty-seven cases of potential polyphyly involving clades of two or more haplotypes were discovered in twenty-three of the participants (Table 1). In all of these cases, the Templeton test strongly rejected the null hypotheses of monophyly (all p's < 10^-4^, the lowest value given by the program PAUP*) despite its conservative bias (see Methods). These conclusions were also confirmed by testing the null hypothesis of monophyly with the Kishino-Hasegawa test, which also yields all p's < 10^-4 ^in PAUP*. Table 1 shows the twenty-three participants (40% of the sample) that satisfied our criteria for multiple infection (see Methods). Of these, eleven participants were inferred to have multiple infection on the basis of polyphyly of *env *alone, eleven on the basis of polyphyly of *pol *alone, and one on the basis of polyphly of both *env *and *pol*. Twenty individuals were inferred to have been multiply infected by just one additional strain, whereas three individuals were inferred to have been multiply infected by at least two additional strains (all had three distinct haplotype clusters in the *env *neighboring joining tree). Out of the 19 participants reporting IDU prior to study baseline, 11 had multiple infections, yielding an incidence of 58% in the IDU subset versus 31% in the non-IDU subset. These differences in incidence between IDU and non-IDU are significant using a one-tailed Fisher's Exact Test (p = 0.045). A one-tailed test is used because of the *a priori *expectation that IDU should increase the risk of multiple infection.

**Table 1:** Patterns of multiple infection in the 23 individuals infected with two or more strains.

Pattern	IDU	Patient ID	Gene	Visit detected	Sampled Visits	No. of Visits Persisted	Max. No. of Possible Visits	Initial Prop.
Co-infected at first visit followed by extinction	0	21	*pol*	1	1,5	1	2	0.80
	0	44	*env*	1	1,6	1	2	0.80
	0	8	*env*	1	1,4,11	1	3	0.20
	1	12	*env*	1	1,3,5	1	3	0.20
	1	49	*pol*	1	1,5	1	2	0.40
	1	50	*pol*	1	1,2,8	1	3	0.40
	1	54	*env*	1	1, 7	1	2	0.20
	1	1*	env*	1	1,10	1	2	0.30
	1	1*	env*	1	1,10	1	2	0.20
	0	58*	env*	1	1,3	1	2	0.20

Co-infected at first visit followed by no detection	0	58*	env*	1,3	1,3	2	2	0.20
	1	19	*env*	1,2,10	1,2,10	3	3	0.60

Superinfected after first visit followed by no detection	0	23	*env*	4	3,4,6	1	2	0.90
	0	38	*pol*	5,8	3,5,8,9	2	3	0.20
	0	10*	env*	3	1,3,5,7	1	3	0.60
	0	10*	env*	3	1,3,5,7	1	3	0.30
	0	10*	*pol*	3	1,3	1	1	0.57
	1	14	*env*	9	1,9,11	1	2	0.50
	1	2	*pol*	3	1,3,6	1	2	0.22

initial infection displaced by a recombinant	0	39	*pol*	7	1,7	1	1	1.00
	0	43	*pol*	3	2,3	1	1	1.00
	0	45	*env*	10	1,5,6,10	1	1	1.00

Superinfected at last visit	0	15	*pol*	4	1,4	1	1	0.20
	0	20	*pol*	9	1,4,9	1	1	0.20
	1	13	*pol*	7	1, 7	1	1	0.50
	1	17	*env*	6	1,6	1	1	0.80
	1	55	*pol*	2	1,2	1	1	0.20

					Average:	1.148	1.963	0.47

*Individuals infected with three or more strains.

The initial proportion is the proportion of the sample at the first visit in which multiple infection was detected that was derived from the second infecting viral strain or, in the case of infections on the first visit, of the strain that was rarest over all visits. Gene symbols marked by an asterisk mean that two additional infecting strains were detected with that gene.

Of the 23 cases of multiple infection, 10 were inferred to be potentially coinfected (infected at the first visit of the study) and 13 definitely superinfected (a secondary infection occurred after an initial infection) (Table 1). There is no significant difference between the incidence of potential co- and superinfection in the total sample. However, IDUs have a significantly higher incidence of potential coinfection than non-IDU's using a one-tailed Fisher's Exact Test (p = 0.035). In contrast, a Fisher's Exact Test of the incidence of superinfection versus no multiple-infection against IDU status was not significant (p = 0.23). Moreover, limiting the analysis to just those individuals with multiple infections, there was no significant association between putative coinfections and superinfections versus IDU status using a Fisher's Exact Test (p = 0.273).

As described in the Methods section, there were no statistically significant differences between IDU and non-IDU in HIV-1 RNA levels and CD4+ cell counts. Similarly, we detected no statistically significant differences in these two variables for multiple versus single infected individuals, superinfected versus non-superinfected individuals, and coinfected versus non-coinfected individuals.

### Temporal patterns of multiple infection

Table 1 summarizes the temporal patterns observed in the 23 participants who had multiple infections. Eight individuals became dual infected on the last visit sampled, thus no inferences concerning the temporal fate of the superinfection can be drawn. However, in three of these eight cases, the only virions detected at the last visit were from the second infection. In the remaining 15 individuals, the evidence for multiple-infection occurred in a visit prior to the last sampled visit, with 10 of the individuals having a multiple infection at the first visit, and hence regarded as potential coinfections. Of the 10 putative coinfected individuals, two were infected with three strains at the first visit. In two of the coinfected cases, the multiple-infection persisted throughout all subsequent visits. Of the 18 strains found in the 15 individuals with multiple infections prior to the last visit (*pol *is excluded from subject 10 because *pol *was not scored on the last visit, although this individual was placed into this class on the basis of *env*, which was surveyed on the last visit), the evidence for the superinfection was lost before the last visit for 16 strains (89%).

The average length of a multiple-infection is 1.15 visits (Table 1), and even when we exclude all participants in which the multiple infection occurred only on the last visit, the persistence time is still a low 1.21 visits.

### Intergenic recombination between strains in multiple-infected individuals and selection on recombinants

Of the 23 individuals inferred to have multiple infections, only one was so inferred by both the *pol *and *env *genes (individual 10, Table 1). Moreover, this individual experienced an additional infection, for a total of three infecting strains, but the third strain was only detected by the *env *gene. Hence, all 23 individuals with multiple infections and 25 out of 26 multiple infecting strains (96%) experienced recombination between the *pol *and *env *genes with the parental types being from two distinct infecting strains. Only one superinfecting strain in one participant had no detectable recombination between *pol *and *env*.

The initial average frequency of the secondary infecting strain (or the strain that is numerically less dominant over all visits when strains coexist during the first visit) is 0.47 (Table 1). This average includes the three cases in which the second infection completely displaced the first infection in our sample. Excluding those cases reduces the average initial frequency to 0.40. Neither of these frequencies is significantly different from 0.5. Hence, the secondary infecting strain initially becomes nearly as frequent as the first infecting strain. Under neutrality, we would therefore expect roughly equal numbers of hosts to lose either the initial strain or the recombinant strain given that one or the other is ultimately lost. Of the 25 strains showing recombination between *pol *and *env *in Table 1, one strain ultimately declined to undetectable levels in 19 cases. Of these, 16 (84%) lost the recombinant strain and 3 (16%) lost the non-recombinant initial strain. Assuming a binomial distribution with *p *= 0.5, a difference that large or larger has a probability of 0.0021 under the null hypothesis of neutrality.

### Intragenic recombination within and between strains in all individuals

Table 2 presents the inferred number of recombinants meeting our criteria to eliminate PCR artifacts (see Materials and Methods) over all individuals studied as a function of IDU status, superinfection status, and gene sequenced. The rates of recombination (number of recombinants divided by number of individuals) vary greatly over these categories. An exact test of homogeneity of intrastrain recombination rates over the 8 distinct categories formed from the combinations of IDU status, superinfection status, and gene rejected the null hypothesis of homogeneity with a 2-sided probability of 0.0001, and similarly the null hypothesis of homogeneity was rejected for the total intra- and interstrain recombination rates with a 2-sided probability of 0.021. There were only 5 confirmed intragenic, interstrain recombinants, which were too few to perform any meaningful tests of homogeneity on that class alone.

**Table 2:** Intragenic recombination events.

IDU	Multiple Infected	Gene	No. Ind.	No. of Intrastrain Recombinants	No. of Interstrain Recombinants	Rate of Intrastrain Recombination/Ind.	Rate of Interstrain Recombination/Ind.	Total rate of Recombi-nation
No	No	*pol*	27	4		0.148		0.148
No	No	*env*	28	28		1.000		1.000
No	Yes	*pol*	11	5	1	0.455	0.091	0.455
No	Yes	*env*	10	7	0	0.700	0.000	0.700
Yes	No	*pol*	7	4		0.571		0.571
Yes	No	*env*	7	21		3.000		3.000
Yes	Yes	*pol*	12	5	1	0.417	0.083	0.500
Yes	Yes	*env*	12	4	3	0.333	0.250	0.58

Numbers of confirmed intragenic recombination events detected are subdivided as a function of the IDU status, superinfection status, and gene sequenced. Recombination events are further divided into those between viruses from the same monophyletic strain within a subject versus those that occurred between strains in superinfected individuals.

To examine the source of this heterogeneity, we performed a logistic regression analysis using the presence or absence of recombination as a binary response variable, weighted either by the number of participants or the number of recombination events given some recombination, with the factors of IDU status, multiple infection status, and gene (*pol *or *env*), and all pairwise interactions among these factors. Because the results were very similar under either weighting scheme, only the results weighted by the number of recombinants when recombination was present are shown. Table 3 shows the results for intrastrain recombination and Table 4 the results for all recombination. If the singleton recombinants that were excluded because they could be PCR artifacts are included in the analyses, we obtained similar, but muted results (results not shown). For the equivalent of Tables 3 and 4, the IDU and Gene variables remain significant, but show higher p-values than those given in Tables 3 and 4, and the significant MI by Gene interaction in Table 3 is no longer significant. This general muting of statistical significance despite increasing the number of recombinants in the analysis is expected if the excluded class largely represents PCR artifacts. Such artifacts would reduce the biological signal, thereby eroding statistical power despite increasing the number of recombination events in the analysis. However, whether or not these singleton recombinants are included or excluded in the analysis, the general pattern shown in Tables 3 and 4 remains the same.

**Table 3:** Factors affecting intrastrain recombination.

	95% Confidence Interval				
Model Term	Estimate	Standard Error	Lower	Upper	2-sided p-Value
Intercept	-1.748	0.5123	-2.752	-0.7441	0.0006439
IDU	1.929	0.7505	0.2307	3.714	0.02293
MI	1.227	0.6779	-0.3029	2.807	0.1315
Gene	2.456	0.5782	1.242	3.842	6.74 × 10^-06^
IDU*MI	-1.64	0.7985	-3.53	0.1774	0.08421
IDU*Gene	-0.8162	0.8053	-2.697	1.05	0.5287
MI*Gene	-1.815	0.7809	-3.637	-0.02093	0.04689

Results of the logistic regression on the binary variable of the presence or absence of intrastrain recombination as weighted by the number of recombinants given some recombination against the factors of injection drug use (IDU) status, multiple infection (MI) status, gene (*pol *or *env*), and all their pairwise interactions. All probabilities are exact.

**Table 4:** Factors affecting all recombination.

	95% Confidence Interval				
Model Term	Estimate	Standard Error	Lower	Upper	2-sided p-Value
Intercept	-1.707	0.5043	-2.695	-0.7184	0.0007132
IDU	1.813	0.7398	0.1353	3.563	0.03189
MI	1.142	0.6716	-0.3765	2.7	0.1626
Gene	2.4	0.5705	1.201	3.761	9.38 × 10^-06^
IDU*MI	-1.269	0.7786	-3.104	0.5017	0.1978
IDU*Gene	-0.635	0.7868	-2.461	1.187	0.6697
MI*Gene	-1.675	0.7693	-3.464	0.09065	0.06576

Results of the logistic regression on the binary variable of the presence or absence of all recombination as weighted by the number of recombinants given some recombination against the factors of injection drug use (IDU) status, multiple infection (MI) status, gene (*pol *or *env*), and all their pairwise interactions. All probabilities are exact.

Of the observed five inter-strain, intragenic recombination events in multiple infected individuals, two were detected at visits other than the visit at which polyphyly was detected (our indicator of multiple infection). In one case (subject 14 in Table 1), the interstrain recombinant was detected in visit 1, the visit sampled just before the next sampled visit (visit 9) at which polyphyly was detected. This indicates that the multiple infection had actually occurred earlier than the visit at which polyphyly was detected. This is not surprising given that our sample sizes were usually 10 per visit, so polyphyly would not be detected with a high probability until the secondary strain had built up its numbers. In the second case (subject 50 in Table 1) polyphyly was detected only at visit 1, but the recombinant was detected at visit 8, two sampled visits removed from the visit leading to the inference of multiple infection. Although all phylogenetic evidence for multiple infection ended by visit 2, the multiple infection obviously had a long-term effect, with some of its genetic material persisting to the last sampled visit.

### Rates of multiple-infection estimated from data subsamples

Table 5 presents the estimated incidence of multiple infection in our total data set and in various subsamples of our data. As can be seen, the expected incidence of multiple-infection is strongly influenced by the sampling design. Table 5 also presents the estimated incidence of multiple-infection from other studies in the row that corresponds most closely to the sampling design used by that study. An arcsin, square root test was also used to test the null hypothesis that the incidence of multiple infection in the other study was the same as the expected incidence in the appropriate subsample of our data. The probability level of the resulting test is also given in Table 5. In three cases, our observed or estimated incidence of multiple infection was not statistically significantly different from that of other studies, in one case the difference was barely significant at the 5% level, and in one case the difference was significant. Because we are testing the same null hypothesis multiple times, we also used a Bonferroni correction for multiple testing. This correction indicates a required threshold of p < 0.010 for overall significance at the 5% level. Only the contrast of our results with Tsui et al. [14] is significant. The most direct comparison between our study and that of Tsui et al. [14] is for the *env *gene, the only locus scored in common in the two studies. Tsui et al. [14] scored between 10 to 13 *env *sequences per subject for six individuals over two visits per subject. Our expected incidence of multiple infection for a similar subsample of our data is 14%. The probability that all six individuals would yield no inference of multiple infection given a 14% expected rate is 0.40. Hence, when a direct comparison can be made, our results are not statistically inconsistent with those of Tsui et al. [14]. More individuals were scored for the first *tat *exon and p17 sequences in the Tsui et al. study, but these genetic surveys were not done from random plasmid subclones, invalidating any further direct comparisons.

**Table 5:** Multiple infection (MI) rates from the total data set and various subsamples.

Sample or Subsample	Observed or Expected Number of MI	Observed or Expected Incidence of MI	Incidence of MI from other study	Sample Size Other Study	p-value	Refer-ence Other Study
All data	23.00	0.40	0.143	7	0.2293	[9]
*pol *data only	12	0.21	0.078	64	0.0431	[12]
*env *data only	12	0.21				
2 visits per subject	19.92	0.34				
2 visits per subject; *env *data only	10.67	0.18				
2 visits per subject; *pol *data only	9.75	0.17				
2 visits per subject; *env *data only; 10 sequences per subject	8.14	0.14	0.000	37	0.0031	[14]
1 visit per subject	8.83	0.15				
1 visit per subject; *env *data only	3.58	0.06	0.013	147	0.0793	[10]
1 visit per subject; *pol *data only	5.25	0.09				
1 visit per subject; *pol *data only; 2.5 sequences per subject	3.60	0.06				
1 visit per subject; *pol *data only; 2.5 sequences per subject; assume no intergenic recombination	0.16	0.00	0.000	718	0.2563	[11]

## Discussion

Because we identified a large sample size of multiple-infected individuals in a longitudinal study, we were able to observe a diverse array of temporal patterns (Table 1). The most common pattern is the rapid elimination of the secondary infecting strains. Hence, the multiple-infected state is largely transitory. Due to our sample sizes of 10 sequences per visit, we cannot completely exclude persistence at a low frequency, though it is obvious that the most common fate is for one strain to become numerically dominant shortly after a multiple infection occurs. All of our subjects were HIV+ when enrolled in the study, and some of them may have had multiple infections prior to enrollment that had been resolved into a homogeneous population by the time of sampling. Also, we would not detect any superinfections that occurred between two visits and that had become resolved prior to the sampling for the second visit. Hence, our estimate of a multiple infection rate of 40% is conservative.

This rapid elimination of the secondary strain is not expected from the initial state of the multiple-infection. As shown in the Results section, the secondary infecting strain initially becomes nearly as frequent as the first infecting strain, but then tends to rapidly lose its numerical parity and becomes undetectable. These dramatic numerical changes imply strong non-random forces. The initial high frequency of the second infecting strain could be explained by an initial escape of the secondary strain from a strong immune surveillance by the host, just the opposite of the immunological protection hypothesis proposed by others [11,13,14]. This initial advantage might then be lost as the host's immune system begins to target the new, numerically co-dominant strain. The subsequent rapid numerical decline of the secondary strain indicates that the first strain has a strong competitive advantage, perhaps due to having had a longer period of evolutionary time in which to adapt to the local host environment. An exception to this pattern is the two cases in which the multiple-infection persisted from the first to the final visit. Both of these cases are possible co-infections, so both strains could have about the same amount of time to adapt to the local host and both could be targeted by the immune system equally. Under the competitive exclusion principle, the two cases of co-infection with continued coexistence could be explained by each strain adapting to different niches within the host and/or by having density-dependent competitive inhibitory interactions with one another [17,18].

In three participants (13%) the original strain was displaced by a secondary strain (Table 1), a pattern previously reported in studies of single superinfected individuals [9,4]. This displacement is only partial in a genetic sense since all three cases of displacement involved an intergenic recombinant. Likewise, previous reports of displacement were due to a recombinant between the initial and the superinfecting strain [9,4]. Thus, the initial strain was not completely replaced genetically, but rather some of its genetic material was used by the displacing superinfecting strain.

Our observed probability of recombination between multiple infecting strains was 0.96, indicating that interstrain recombination is common in multiply infected individuals, as expected from previous studies [1,2,8]. The high frequency of recombinants does not necessarily mean that recombinants are selectively favored; indeed, our results revealed significant overall selection against interstrain-recombinants (the null hypothesis of neutrality is rejected with a probability of 0.0021). Hence, most of the time, selection appears to work to eliminate the superinfecting strain and its recombinants, but occasionally some recombinants may have very superior fitness [13], as shown by our three cases of recombinant displacement.

We detected 78 intrastrain recombination events and five interstrain recombinants in multiple infected participants (Table 2). The intrastrain recombination reveals many non-random patterns, as shown by the homogeneity and logit (Tables 3 and 4) analyses. First, injection drug users experience significantly higher levels of recombination (Tables 3 and 4). Second, the *env *gene displays more recombination than the *pol *gene despite the fact that the average length of the *pol *sequences in our study was 1496 bp versus 686 for *env*. One possible explanation is that there is more recombination within *env *than within *pol*, but the opposite has been observed using an *in vitro *recombination system [19]. Hence, either the *in vivo *recombination patterns are different from those *in vitro*, or another factor is operating that reverses this recombination bias. This other factor may be selection. We only score as recombinants those recombination events that left two or more descendants, and therefore have demonstrated at least a minimal degree of evolutionary success. Our previous studies indicate strong positive selection on the *env *gene within these same individuals [20]. Intragenic recombination within *env *could be an important source of variation upon which this selection could operate, thereby amplifying the apparent amount of intragenic recombination within *env *despite a recombination bias in favor of *pol *at the molecular level [19].

In two cases inter-strain, intragenic recombination events were detected at visits after the visit at which polyphyly was detected. These persistent recombinant genetic materials were not detected as a continuation of the multiple-infection because the section of the genes that came from the secondary strain was so small that the recombinant clustered with the primary infecting strain to form a single monophyletic group in the neighbor joining tree. Thus, if only these latter visits had been sampled, the criterion of polyphyly, which is standard in this literature, would have failed to detect any evidence for multiple-infection even though that evidence was present in the multi-visit analysis. Thus, the criteria of polyphyly alone can fail to detect multiple infections that have been affected by much recombination.

In light of these biases, we conclude that multiple-infection is common, but difficult to detect because natural selection and/or competitive exclusion causes the multiple-infected state to be highly transitory. The one lasting legacy of such multiple infections is recombinant virions. Most recombinants do not survive long in the host, but a few persist throughout the infection, and some of these recombinants even displace the original infection, indicating superior fitness and competitive ability. The pattern observed in our cohort is compatible with the observation that recombinant clades of HIV-1 are common throughout the world. Thus, multiple infection and recombination significantly add to the genetic diversity of HIV-1 and its evolutionary potential, and injection drug use significantly increases both.

## Conclusion

Our multiple infection incidence of 40% is consistent with the inference of high rates of multiple infection from inter-subtype recombination data [1,8], but it is significantly higher (a 2-sided p-value of 1.4 × 10^-5^) than the indirectly estimated intra-subtype multiple infection rate of 15% [8]. This discrepancy is explicable due to the significant selection we detected against the interstrain recombinants. Our rate of successful superinfection recombinants is between 5/58 (9%) (individuals with superinfections that survived to the last visit but appeared in earlier visits) to a maximum of 10/58 (17%) (by adding in those individuals who became superinfected on the last visit), a range that straddles the indirect estimate of 15% [8]. Hence, our results explain well the rate at which such recombinants are detected in the general HIV-1 population.

Our multiple-infection incidence of 40% is not statistically significantly different from the direct incidences between 0–14% reported in previous studies (Table 5 and Results section), illustrating the critical importance of sampling design in making inference. Hence, there is no real discrepancy between the direct and indirect estimates of multiple-infection incidence.

The fact that our cohort had a high incidence of multiple infection, and specifically superinfection, undermines the hypothesis that an initial HIV-1 infection produces some degree of protection against superinfection [11,13,14]. This in turn may imply that vaccine development will be difficult, as indeed appears to be the case [21,22]. However, these superinfections occur, at least in part, in individuals whose immune systems have already been compromised by HIV, a situation that will not pertain to vaccinated individuals. Hence, our results do not mean that an effective vaccine cannot be developed, but rather they do caution us about the difficulties of vaccine development.

## Methods

### Study population

The Women's Interagency HIV Study (WIHS) is a multicenter, prospective cohort study to investigate the impact of HIV-1 infection on women [23]. In 1994, 2,628 women (2,059 HIV-1 positive and 569 HIV-1 negative) were recruited by both institution and community based programs. Every six months the participants met with study personnel for an encounter termed a "visit", during which WIHS participants are interviewed using a structured questionnaire and received a physical examination [23]. Informed consent was obtained from all study participants at the individual WIHS sites and human experimentation guidelines of the individual sites and of the Johns Hopkins Bloomberg School of Public Health were followed in the conduct of this research.

Fifty-eight HIV-1 infected individuals contributing 123 study visits were selected for analysis. All samples were from visits that occurred between initiation of the WIHS and 2000. All participants met the following criteria: 1) A defined IDU status 2) a visit within 12 months prior to initiating highly active antiretroviral therapy (HAART) 3) a viral load >4,000 copies/ml of plasma to avoid re-sampling the same virion [24] and 4) a CD4 T cell count <200 on the last pre-HAART visit as an indication of disease progression. Nineteen IDU (33%) met these criteria and from the non-IDU that met the criteria 39 (67%) were randomly selected for further analysis.

The median age of the 58 WIHS participants at baseline was 38 years, the overall median log_10 _(HIV-1 RNA) level was 4.80 cps/ml and the overall median CD4+ cell count was 311 cells/mm^3^. The majority (64%; n = 37) of study participants were African-American. Compared to the non-IDUs, IDUs had higher median log_10 _HIV-1 RNA levels (4.97 (4.40, 5.34) vs. 4.66 (4.15, 5.26)) and lower median CD4+ cell counts (200 (85, 479) vs. 359 (133, 572)), but the differences were not statistically significant. Racial composition did not differ between the IDU and non-IDU groups. Study participants reporting a history of IDU were older than those not reporting IDU prior to enrollment (40 vs. 35; P = 0.03). All participants reporting IDU were HCV positive at baseline, while only 4 (11%) of the non-IDUs were HCV positive (P < 0.01). Although treatment was initiated from different sites within the multi-centered WIHS cohort, treatment was generally based on the standard of therapy at the time of each subject's study visit. Among non-IDUs, 20 (51%) participants reported using monotherapy or combination therapy prior to study entry, compared to 12 (63%) participants with a history of IDU (P = 0.72). All monotherapy and combination therapy reported prior to study enrollment consisted of only nucleoside and/or non-nucleoside reverse transcriptase inhibitors.

### Sequencing technique

A total of 1100 cloned sequences of the *pol *gene and 1100 of the *env *gene of HIV-1 were obtained as described in an earlier study [25]. Additional sequences were obtained for this study to fill in some sampling gaps, for a total of 1,127 *pol *and 1236 *env *sequences. Our goal was to sample 10 sequences for each gene from each visit (1230 total sequences for each gene given 123 visits), but occasionally that goal was not meet, with the smallest sample size per gene per visit being four. HIV-1 RNA was isolated from stored samples of plasma using the QIA amp viral RNA mini-kit (QIAGEN, Valencia, California, USA). The isolated RNA was subjected to RT-PCR (Life Technologies Superscript One-Step RT-PCR for long templates). To avoid contamination among subject visits, all plasma samples from a subject visit were processed for reverse transcription and amplification singly (one at a time) in a PCR clean room within the laboratory in which no amplified specimens were permitted. After sequencing, all sequences from the study population were aligned and placed on a single phylogenetic tree to ensure that there were no closely related sequences appearing among different individuals. In eighteen instances (out of the 2364 total sequences) an *env *or *pol *sequence was indeed phylogenetically located within a monophyletic cluster defined by the sequences from a different subject. All eighteen sequences were regarded as potential contaminants and excluded from all subsequent analyses.

For the *pol *gene, we used the primers pro-1 (TTGGAAATGTGGAAAGGAAGGAC) and RT-0 (CATATTGTGAGTCTGTTACTATGTTTAC) with cycles of 50°C 30 minutes, 94°C 2 minutes, and 35 cycles of 94°C 40 seconds, 50°C 40 seconds, 68°C 3 minutes, followed by one cycle of 72°C 10 minutes and then held at 4°C. A second round PCR was run using the Gene Amp XL PCR kit (Roche Applied Biosystems, Indianapolis, IN), with the primers pro-3 (GAGCCAACAGCCCCACC) and RT-3 (GCTGCCCCATCTACATAGAA); with an amplification protocol of 94°C for 1 min, followed by 35 cycles of 94°C for 40 seconds, 52°C–56°C for 40 seconds, 68°C for 2 minutes, 30 seconds, followed by one cycle of 72°C for 10 minutes with the product held at 4°C until it was harvested and run on an 8% agarose gel. A band at the 1,617 base-pair size was extracted from the gel using the QIA Quik Gel Extraction Kit (Qiagen, Valencia, California, USA), and the obtained DNA was ligated into the TOPO 2.1 vector and transformed into TOPO 10 competent cells (Qiagen, Valencia, California, USA), according to the manufacturer's instructions. The transformed cells were plated on LB agar plates containing 50 μg/ml Ampicillin and 40 μl of 40 mg/ml X-gal. Confirmed transformants were grown overnight and plasmid DNA was extracted for sequencing, using an ABI prism 3700 DNA Analyzer (Perkin Elmer Biosystems, Boston, Massachusetts, USA). The cloned sequences were obtained in nucleotide format and translated into amino acids using MegAlign software by DNAStar (DNASTAR Inc., Madison, WI). The entire protease (*PR*) region (297 nucleotides) and partial reverse transcriptase (p*RT*) region (674 nucleotides, including all known sites of resistance mutations) were available from each of the 123 study visits [25]. The *pol *sequences generated are available through Genbank, Accession Numbers EF374379–EF375478. Note that these sequences were aligned for each individual subject, but were not aligned across individuals. Phylogenetic analysis requires aligned sequences, both within and across individuals, and a file containing the alignment for all *pol *sequences is available upon request from ART.

The same technique was used for sequencing the C2–V5 regions of the *env*gene. The first round primers were ED12C (AGTGCTTCCTGCTGCTCCCA) and ED31C (CCATTACACAGGCCTGTCCAAAG) and the second round primers used were DR7C (TCAACTCAACTGGTCCAAAG) and DR8C (CACTTCTCCAATTGTCCCTCA) that yield data on 694 nucleotides in the aligned sequences. The *env *sequences generated are available through Genbank, Accession Numbers EU040366–EU041600. Note that these sequences were aligned for each individual subject, but were not aligned across individuals. Phylogenetic analysis requires aligned sequences, both within and across individuals, and a file containing the alignment for all *env *sequences is available upon request from ART. Because the sequences are very similar within the monophyletic clusters, our principal concern was the alignment across clusters. To check the quality of this alignment, representative sequences were chosen from the monophyletic clusters and assessed for alignment quality using the program ClustalX [26]. For *pol*, the low quality sites were highly scattered, indicating an overall excellent alignment with no problematic blocks. For *env*, there were two clusters of low quality alignment, one of 29 nucleotides in length and a second of 18 nucleotides in length. Both regions were characterized by many inferred insertions or deletions. The inclusion or exclusion of these nucleotide sites had no impact on the topology of the neighbor-joining tree relative to the inferred monophyletic clusters, the only purpose for which this tree was used. The *env *and *pol *neighbor-joining trees are available in additional files 1 and 2.

### Inference criteria for multiple, coinfection and superinfection

All the *pol *sequence data from all participants and all visits were used to construct a neighbor-joining tree for the *pol *gene using PAUP* [27], and likewise all the *env *sequence data from all participants and all visits were used to construct a neighbor-joining tree for the *env *gene. The program ModelTest [28] was used to fit the nucleotide data to a substitution model, and for both *env *and *pol*, the best fitting model using the Akaike criterion was TVM+I+G (a transversional model with unequal base frequencies, some invariant sites, and rate variation among sites). Our only use of these neighbor-joining trees was to test for monophyletic clusters. As to be described, all the monophyletic clusters in these data were separated by multiple mutations (a minimum of 31) that yield extremely long branch lengths in the neighbor-joining trees that would be easily detected by any clustering technique. As will also be described, we did not use neighbor-joining to infer the evolutionary trees within a monophyletic cluster but rather used the Bayesian procedure of statistical parsimony.

An individual subject was regarded as having only a single source infection if both the *pol *and *env *sequences defined a single monophyletic cluster in the respective multi-subject neighbor-joining trees. Additional analyses were performed if one or both genes from a specific subject defined two or more disjoint clusters (polyphyly) within the multi-subject neighbor-joining tree(s). When polyphyly was detected, a tree was constructed that forced all the sequences from a single subject to be monophyletic, and the Templeton test option [29,30] in PAUP* [27] was used to test the null hypothesis that the polyphyletic tree was not significantly different from the monophyletic tree. When sequences are forced to be monophyletic, long branches are created in the trees to explain the enforced monophyly. Homoplasy (multiple mutational hits at the same nucleotide site that cause reversals and/or parallelisms) are very common in HIV data, and long branches tend to be underestimated in length preferentially by parsimony when homoplasy is common. Because the Templeton test acquires greater statistical power as the estimated branch length increases, the high levels of homoplasy typical of HIV data sets means that the Templeton test will be a statistically conservative test of monophyly.

As discussed previously, 18 sequences were regarded as possible contaminates and excluded from this analysis of polyphyly. Multiple infection was inferred only when two or more distinct polyphyletic clades (branches) existed within an individual such that at least two clades contained two or more haplotypes for one or both genes.

Multiple infections detected on the first visit were regarded as potential coinfections, and all other cases of multiple infection were regarded as superinfections. As all of the participants were already HIV positive at baseline, it is possible that some of the potential coinfected cases were actually superinfections. Hence, our estimate of coinfection may be biased upwards and our estimate of superinfection may be biased downwards. This also means that all tests of heterogeneity between coinfected and superinfected individuals will be biased in favor of the null hypothesis of homogeneity.

### Recombination

Recombination between the *pol *and *env *genes in multiple-infected individuals was inferred when only one of these genes resulted in polyphyly. Recombination within the *pol *sequences and within the *env *sequences was inferred by the method of Crandall and Templeton [15] as modified by Templeton et al. [16]. This method was specifically developed for detected recombination in HIV [15]. Separate evolutionary trees for the *pol *and *env *sequences of all the haplotypes (unique sequences) found in a single individual over all visits were estimated using statistical parsimony [31] with the program TCS [32]. The haplotype tree represents the null hypothesis of no recombination. Individual mutational transitions that appear on multiple branches (homoplasies) in the tree may be the result of recurrent mutation or recombination. Recombination as a cause of homoplasy can be distinguished from recurrent mutation because homoplasies caused by recombination are physically clustered in the sequence. This results in spatially contiguous runs of homoplasies in the tree. A runs test [implemented in a Mathematica [33] program available by request from ART] is used to test the null hypothesis of no association between homoplasies and physical location in the DNA or RNA region. Recombination is only inferred when the runs test is statistically significant at the 5% level or less. This procedure identifies both the putative recombinant and its parents and localizes the interval in which recombination occurred. This test is particularly appropriate for HIV sequence data, which is strongly affected by mutational homoplasy and selection. The run test is conditioned upon the topology of the tree and depends only upon the clustering of homoplasies on a single branch that are also physically clustered in the nucleotide sequence. The selection that has been documented in HIV sequence data is not associated with such close physical clustering [20], and most tests of selection are sensitive to frequencies of SNPs or haplotypes, which do not enter into this statistic at all. Moreover, high levels of homoplasy often cause loops in the statistical parsimony tree, which represent phylogenetic ambiguities. However, when tracing runs through such loops, the resulting set of runs is invariant to how the loop is traversed and depends only upon the nucleotide differences between the sequences at the end-points of the run.

RT-PCR can also induce recombination during sequence amplification [34]. To focus only on recombination events that occurred naturally within an infected subject, we excluded all those recombination events that were identified by only a single recombinant sequence, which always had to be located at the terminus of a branch in the evolutionary tree of haplotypes. We regarded as true recombination only those events from which a monophyletic branch (clade) evolved that contained two or more sequences in the evolutionary tree of haplotypes.

### Statistical analyses

The null hypothesis of no association between two binary categorical variables was tested with a Fisher's Exact Test, as implemented in the program StatXact 7.0 (Cytel Software Corporation). Homogeneity of recombination rates over various classifications was also tested with an exact test with StatXact 7.0. An exact logistic regression was performed with the program LogXact 7.0 (Cytel Software Corporation) to investigate the impact of IDU status, multiple infection status, and gene upon recombination.

Differences in proportions were tested with an arcsin, square root transformation corrected for small sample size [35] as implemented in a Mathematica [33] program available by request from ART. Comparisons between various groups of participants for viral load and CD4+ cell counts were executed in Excel (Microsoft) using a two-tailed t-test without assuming equal variances.

Because our sample design is fuller than that of most previous surveys for multiple infections, we also analyze subsamples of our data in order to compare our results to previously published results. In some cases our subsample is based on a stratifying variable, such as a subsample based upon having only *pol *sequence data. In such cases, we simply estimate the rate of multiple-infection from our data using only the information gained from *pol *data strata and ignoring the *env *sequence data strata. In other cases, we form a subsample at random. For example, to simulate what we have found if we only had cross-sectional data, we calculate the rate of multiple infection that we would have observed by using the data from only one randomly chosen visit per subject. Other subsamples reflect a mixture of these stratifying and random subsamples; e.g., a sub-sample that simulates a cross-sectional study done only with *pol*.

## Competing interests

The authors declare that they have no competing interests.

## Authors' contributions

ART executed all of the phylogenetic, some of the recombination analyses, and all of the remaining statistical analyses. MGK and JJ executed most of the recombination analysis. SG and MS were responsible for collection, maintenance and analysis of subject data, including CD4 and viral load levels and resistance patterns. GWZ and QS performed sequencing. JK, M-FY, H-LT, and HZ were involved in organization and analysis of sequence data. RM initiated studies of these populations for sequence analysis and has discussed the theoretical basis for these studies extensively with ART. Dr. Markham's laboratory performed all of the sequencing required for these analyses.

## Supplementary Material

Additional file 1 ***env *sequence data**. 1226 sequences of HIV-1 *envelope *genes at a total of 694 nucleotide sites (including deletions inserted for alignment). Click here for file

Additional file 2 ***pol *sequence data**. 1127 sequences of HIV-1 *pol *genes at a total of 1620 nucleotide sites (including deletions inserted for alignment). Click here for file
